# Erdheim-Chester disease, moving away from the orphan diseases: A case report

**DOI:** 10.1016/j.rmcr.2016.11.013

**Published:** 2016-12-03

**Authors:** Jessica M. Stempel, Jean G. Bustamante Alvarez, Andres Mora Carpio, Varun Mittal, Claudia Dourado

**Affiliations:** a Albert Einstein Medical Center, Department of Internal Medicine, Philadelphia, PA, USA; b Albert Einstein Medical Center, Department of Hematology and Oncology, Philadelphia, PA, USA

**Keywords:** Erdheim-Chester disease, BRAF V600E mutation, Vemurafenib

## Abstract

With approximately 750 cases reported, Erdheim-Chester disease is an exceedingly rare histiocyte cell disorder. Affected sites typically include long bones, large vessels and central nervous system. However, cutaneous and pulmonary involvement can also occur. The diagnosis is ascertained by identification of foamy histiocytes positive for CD68, CD163, and factor XIIIa on immunoperoxidase staining. Recently published literature have described an association between Erdheim-Chester disease and BRAF V600E mutation. This finding prompted the investigation of therapeutic possibilities with BRAF inhibitors, successful agents against other *BRAF* mutation-positive diseases. Vemurafenib, a BRAF kinase inhibitor, has been shown to be effective in *BRAF* V600E mutation-positive malignancies, such as NSCLC and melanoma, as well as in several case reports of Erdheim-Chester disease. We report a case of Erdheim-Chester disease diagnosed at our institution, treated with vemurafenib.

## 1 Case report

**Fig. 1:**
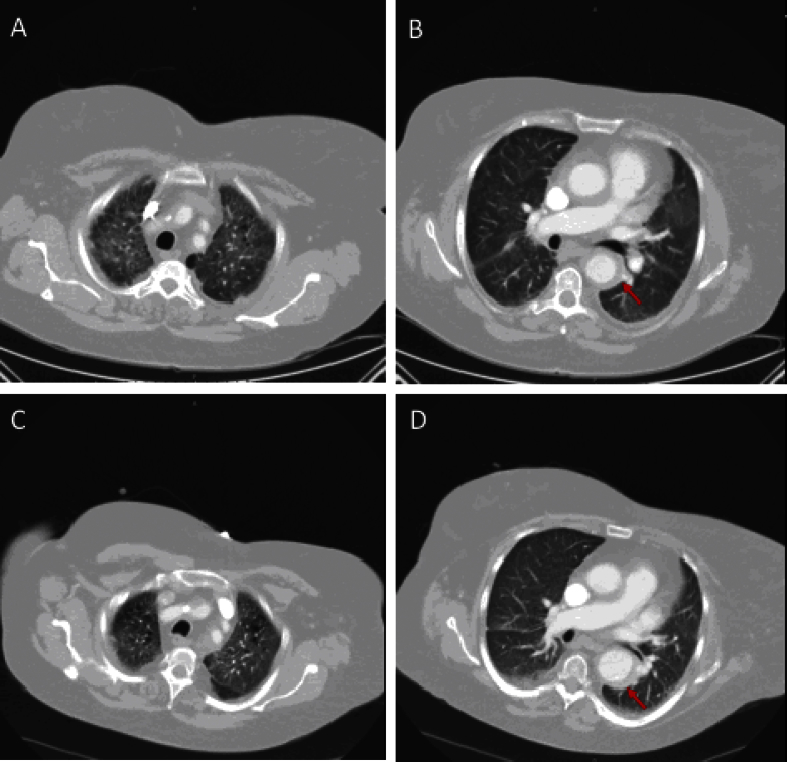
**Panel A and B.** Initial CT demonstrating typical peri-aortic thickening, ground-glass opacities as well as the presence of cysts. **Panel C and D.** Follow-up CT chest shows stable disease following 6 months of treatment with vemurafenib.

**Fig. 2:**
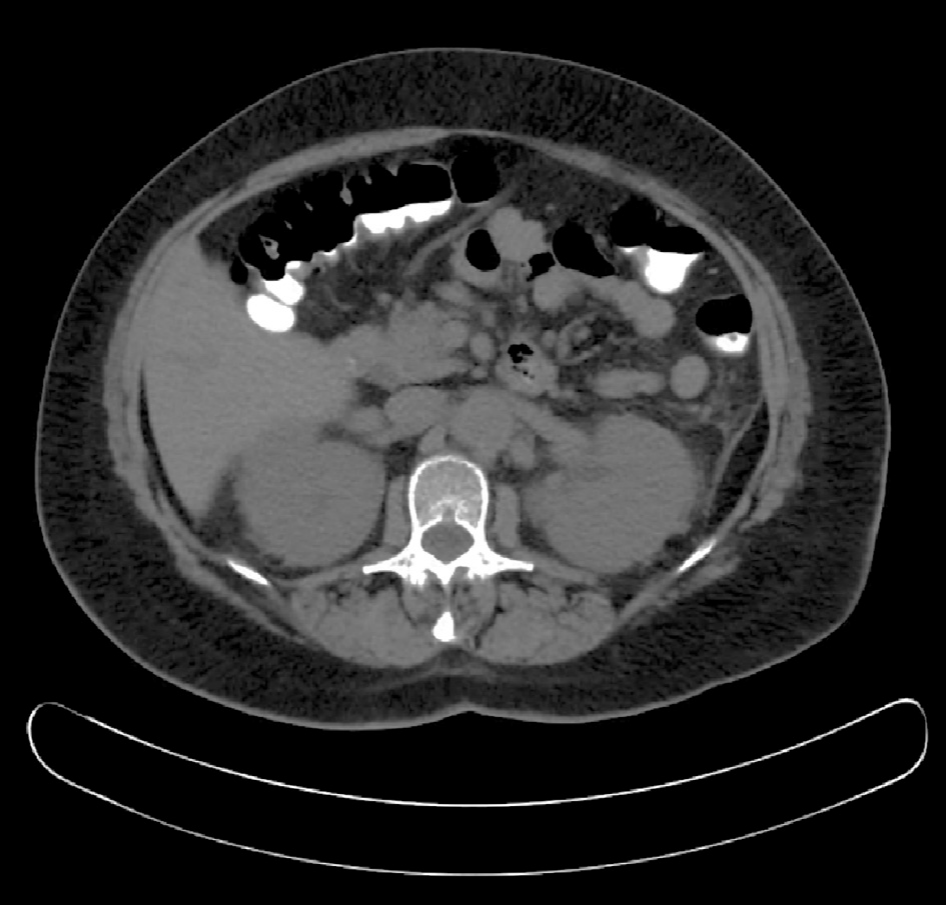
**Abdominal CT** demonstrating perinephric infiltrate bilaterally, giving a “hairy kidney” appearance.

We present a case of a 58-year-old female who presented with progressively worsening exertional dyspnea and non-productive cough. She reports a past history of tobacco use. On initial examination, periorbital xanthelasmas and diffuse wheezing were noted and she was admitted for presumed chronic obstructive pulmonary disease exacerbation and her symptoms improved with standard therapy. The patient's chest X-ray demonstrated diffuse reticular opacities. A chest computed tomography (CT) demonstrated ground-glass opacities, peripheral cysts, and aortic wall thickening, as well as non-specific vertebral sclerosis and kidneys with “hairy” appearance (Fig. 1, Fig. 2). During the following four months, the patient had progressive dyspnea, requiring supplemental oxygen (4 L/min). Given her worsening respiratory symptoms and hypoxemia, a lung biopsy was performed. Immunoperoxidase staining of the biopsy sample revealed antibodies against factors XIIIa and CD68, and was positive for *BRAF* gene mutation by PRC, V600E (1799 T > A). These clinical findings were consistent with Erdheim-Chester disease (ECD). The patient was started on vemurafenib (960 mg twice daily) with interval improvement in respiratory symptoms and decrease in oxygen requirements (2 L/m) over approximately 4 months. A CT scan of the chest was repeated approximately 6 months following initiation of therapy, and demonstrated improved, albeit persistent radiologic evidence of pulmonary involvement (Fig. 1).

## 2 Discussion

Malignancies in the setting of *BRAF* V600E mutation have rapidly gained interest in part due to the therapeutic availability of BRAF inhibitors such as sobrafenib, dabrafenib and most recently vemurafenib. Erdheim-Chester disease (ECD) or non-Langerhans cell histiocytosis is a rare histiocyte cell disorder with approximately 750 reported cases in the literature since its initial description approximately 85 years ago. The median age of diagnosis is 53 years, with few cases reported in children. There is an increased male prevalence described in the literature—approximately 62% of cases in one systematic review [1].

The etiology of this disease remains unclear. It is suggested that ECD is mediated by a Th1 cellular response by activation of a cytokine-chemokine network, unique to ECD [2]. Moreover, recurrent findings of oncogenic mutations, predominantly *BRAF* V600E, suggests that this disease is a neoplasm of myeloid origin and may occur via the Ras/Raf/MEK/ERK pathway.

The most common clinical findings associated with ECD involve long bones, retroperitoneum, large vessels and the central nervous system, however up to 50% of patients are initially asymptomatic. Although skeletal involvement was unremarkable in our patient, it remains the predominant feature of ECD [3]. Positron emission tomography/computed tomography (PET/CT) of skeletal structures and brain are the preferred test to evaluate burden of disease [4]. Pericardial infiltration and aortic wall coating are common cardiovascular findings. Multiple heterogeneous neurological manifestations have been described in ECD and occur in approximately 51% of cases, ranging from headaches to focal neurological deficits and diabetes insipidus [1], [2]. The presence of CNS disease is a clinical predictor of poor outcome in patients with ECD and should prompt rapid initiation of therapy [5]. Pulmonary symptoms are described in 24% of patients—cough, dyspnea—and approximately 55% of patients have pulmonary involvement on CT imaging [6], [7]. Typical CT findings on lung parenchyma include non-specific ground-glass opacities, septal thickening, centrilobular nodular opacities, and lung cysts, as described in our patient (Fig. 1) [7].

The diagnosis of ECD remains challenging and is based on histopathologic examination of involved tissue, supported by the presence of CD68, CD163 and factor XIIIa on immunohistochemical staining [8]. Chasset et al., described the plausibility of histological diagnosis from skin lesions (xanthelasmas-like lesions) for diagnosis of ECD, highlighting a viable sampling-site requiring a less invasive approach. Additionally, they described the *BRAF* V600E mutation was more frequently detected in patients with cutaneous manifestations of ECD [9].

The rarity of ECD hinders the execution of large randomized trials to assess optimal therapy (Table 1) [10]. To date, there is no consensus on the standard treatment. Interferon-α (IFN-α) has historically been the preferred initial therapeutic modality [8]. Anakinra is an IL-1-receptor antagonist occasionally used as a second-line agent in patients with ECD [11]. Patients with contraindications for IFN-α benefit from alternate therapeutic agents. The use of steroids has not been proven to contribute to survival in patients with ECD. Approximately 54–100% patients with ECD express the *BRAF* V600E mutation [12]. Treatment for ECD is now moving toward targeted therapy mostly due to the high percentage of proven *BRAF* V600E-positive cases. Vemurafenib was approved by the FDA in 2011 for the treatment of *BRAF* V600E-mutation positive, non-resectable and advanced stage malignant melanoma. However, clinical trials are currently ongoing regarding the effectiveness of vemurafenib and other BRAF inhibitors in ECD. Prior case reports have reported treatment success with vemurafenib, with rapid improvement of symptoms and a tolerable side effect profile [13], [14]. Other agents have also been used with success in the treatment of ECD. Cobimetinib, a MEK inhibitor, was associated with clinical improvement in one case series. This case series described treatment response in three patients after clinical progression of disease despite initial therapy, including vemurafenib [15].

As mentioned above, BRAF inhibitors are largely used for treatment of malignant melanoma, another *BRAF* V600E mutation-positive malignancy. Resistance to these agents in the setting of melanoma may develop despite evidence of an observed initial clinical response [16]. Several mechanisms of drug-resistance have been described in patients treated for metastatic melanoma. Vemurafenib resistance is mainly acquired by ERK reactivation via bypass signaling of other proteins in the Ras/Raf/MEK/ERK pathway. Up-regulation of PDGFR-β and NRAS mutations are demonstrated mechanisms of vemurafenib resistance in melanoma-positive samples [17]. Other escape mechanisms include overexpression of COT and CRAF in the MAPK pathway [18], [19]. Secondary mutations in MEK1, downstream from RAF, are also responsible for BRAF and MEK-inhibitor resistance [20]. The rate of BRAF-inhibitor resistance is unknown in patients with ECD. We expect more information will become available once further case reports, case series and clinical trials continue to arise. We anticipate that the above mechanisms of resistance will surface once vemurafenib is more widely used for treatment of ECD, and dual BRAF and MEK-inhibitor strategies will likely be required for patients who develop refractory disease.

All efforts should be made to perform mutational testing on patients diagnosed with ECD. Several clinical trials are attempting to assess the therapeutic benefit and safety profile of vemurafenib and other agents—either alone or in combination therapy—for treatment of ECD [10]. Enrollment in an active clinical trial should be strongly encouraged in patients diagnosed with *BRAF* mutation-positive and negative ECD.

## Conflict of interest

The authors have no potential conflicts of interest to disclose.

## Figures and Tables

**Table 1:** Current clinical trials for Erdheim-Chester disease [10].

Title	Study design	Primary outcome measure	Clinical trial identifier
Long-term Outcome after Vemurafenib/BRAF Inhibitors Interruption in Erdheim-Chester Disease (LOVE)	Prospective, observational cohort	Response assessed by PET scan imaging	NCT02089724
A Phase II Therapeutic Trial of the Use of Dabrafenib and Tramafenib in Patients with BRAF V600E Mutation Positive Lesions is Erdheim Chester Disease	Interventional, open-label trial	Efficacy and safety, response rate, progression and survival	NCT02281760
A Phase II Study of Lenalidomide for Adult Histiocyte Disorders	Interventional, open-label trial	Determine response rate	NCT02523040
Open-label, Single-arm, Phase II, Prospective Pilot Study on Tocilizumab in Patients with Erdheim-Chester Disease	Interventional, open-label, safety and efficacy study	Reduction in measurable lesions by RECIST criteria; functional and quality of life improvement; analysis of AEs, FDG-PET imaging variations.	NCT01727206
Clinical and basic investigations into Erdheim-Chester disease	Observational	Collect study samples and medical information on patients with ECD	NCT01417520

Abbreviations: PET: positron emission tomography; RECIST criteria: Response Evaluation Criteria in Solid Tumors criteria; AEs: adverse events; FDG-PET: Fluorodeoxyglucose positron emission tomography; ECD: Erdheim-Chester Disease.
